# Turning walking pneumonia into recurrent abscesses: a curious case of CVID and review of the literature

**DOI:** 10.1186/s13223-022-00673-3

**Published:** 2022-04-05

**Authors:** David X. Gao, Habiba Hussain, Brianna Bobber, Peter Phan

**Affiliations:** University of Illinois College of Medicine, One Illini Drive, Peoria, IL 61605 USA

**Keywords:** Common variable immunodeficiency, Mycoplasma

## Abstract

**Background:**

Common variable immunodeficiency (CVID) is a primary immunodeficiency disorder associated with a broad symptom presentation that is still being characterized. We report a rare case of recurrent mycoplasma skin abscesses in a patient with a history of autoimmune disorders and prolonged mycoplasma pneumonia who was diagnosed with CVID.

**Case presentation:**

A 34-year-old woman presented with a history of recurrent abscesses previously confirmed positive for *Mycoplasma pneumoniae*. Her past medical history of recurrent mycoplasma abscesses, prolonged mycoplasma pneumonia, and autoimmune disorders (mixed connective tissue disease and immune thrombocytopenia) raised suspicion of CVID. Workup included negative anti-mycoplasma antibody titers, hypogammaglobulinemia, and negative anti-pneumococcal antibody titers despite prior vaccination, solidifying the diagnosis of CVID. The patient was discharged on antibiotic and intravenous immunoglobulin therapy and now follows allergy and immunology long-term for treatment.

**Conclusions:**

Her diagnostic history underscores the importance of considering the various criteria of CVID for diagnosis, and her unique presentation of *M. pneumoniae* skin abscesses highlights the broad sequelae patients with CVID can manifest.

## Background

Common variable immunodeficiency (CVID) describes a syndrome of hypogammaglobinemia (immunoglobulin G [IgG], immunoglobulin A [IgA] ± immunoglobulin M [IgM]) and impaired humoral response to antigens. Multiple mechanisms for CVID have been described, including defects in immune recombination, somatic hypermutation, tolerance, and of B lymphocyte differentiation, in absence of other known immunodeficiency. Due to their deficient immune system, patients with CVID have an increased risk of recurrent respiratory infections, abscesses, pulmonary granulomas, inflammatory bowel disease, autoimmune conditions, and malignancy [1]. However, there is no report to our knowledge on skin abscesses caused by mycoplasma. Herein, we report a case of mycoplasma skin abscesses in a patient whose past medical history raised suspicion for CVID. Her case provides a reminder of the broad diagnostic criteria and clinical sequelae patients with CVID manifest, and we aim to provide a useful summary of the diagnostic criteria, differential diagnosis, and treatment of CVID. We also provide information on this novel symptom of mycoplasma infection in patients with CVID, as well as in the general population.

## Case presentation

A 34-year-old woman with past medical history of mixed connective tissue disease (MCTD) previously on immunosuppressive therapy and immune thrombocytopenic purpura (ITP) status-post splenectomy presented with sepsis secondary to recurrent neck abscesses.

**Fig. 1:**
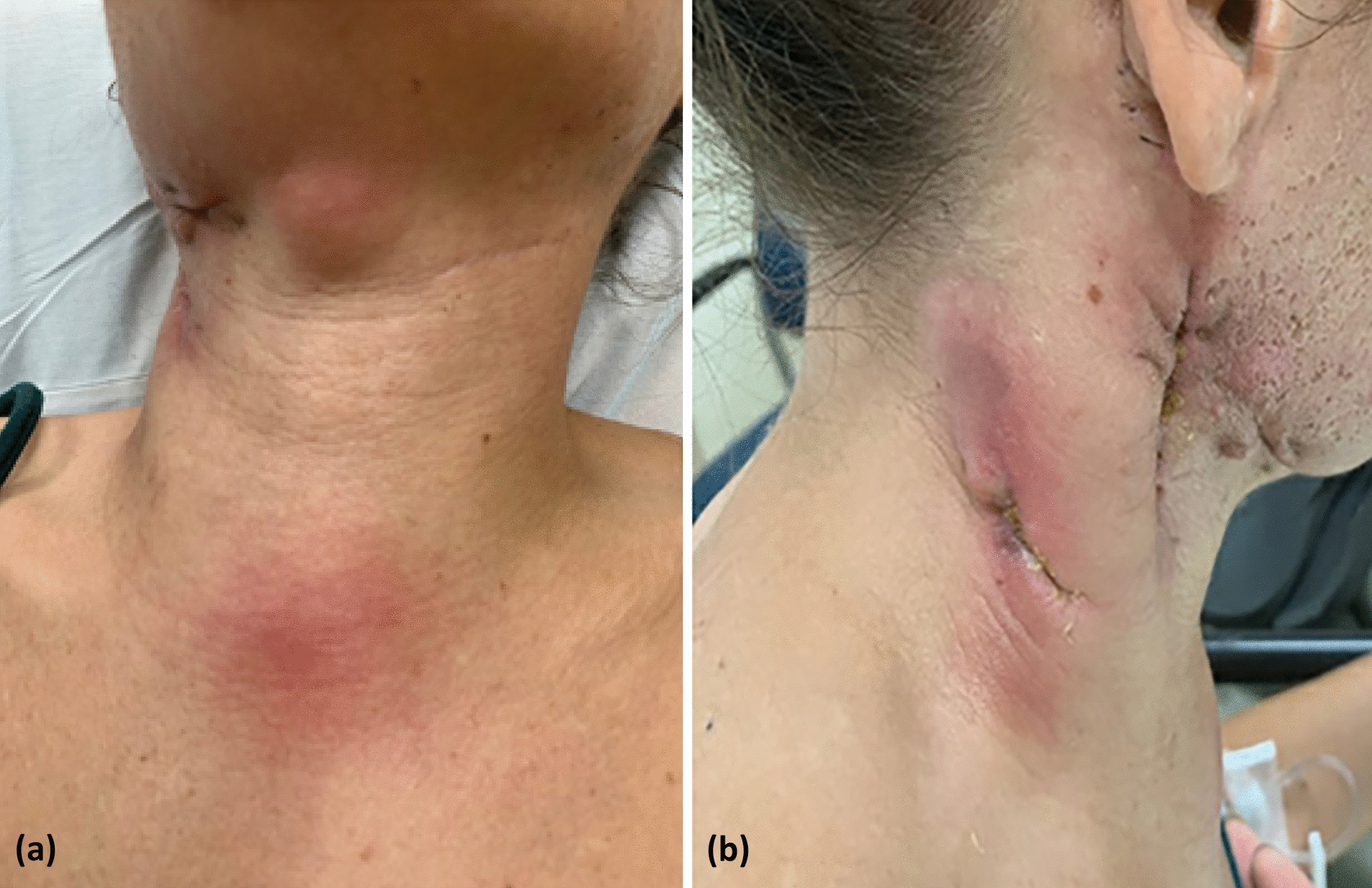
(Left to right): **a** anterior neck abscess prior to incision and drainage. **b** Lateral neck abscess status-post incision and drainage

The patient reported two erythematous, painful, warm, and indurated areas on her chest and posterior neck that had been worsening over the past 10 days (Fig. 1). She had a history of recurrent presentation of neck abscesses over the few years prior to this admission and most recently had a neck abscess drained one month prior. Bacterial culture from the drainage was noted to be negative, but *Mycoplasma pneumoniae* was detected upon microbial cell-free DNA analysis. She was started on oral azithromycin, which she had been taking daily until admission. No evidence of fever, dysphagia, or arthralgia in this time was noted.

**Fig. 2:**
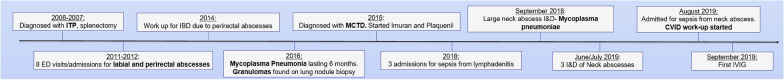
Timeline of patient’s workup

In addition to ITP, past medical history revealed several years of recurrent abscesses of the neck and perianal region with negative cultures. She had previously received rituximab for six weeks over a decade prior to presentation. In 2016 she had mycoplasma pneumonia lasting six months (Fig. 2). She also was found to have pulmonary nodules which were biopsied, revealing granulomatous inflammation. Later that year, due to her history of ITP, pulmonary nodules, positive antinuclear antibody (ANA), positive ribonucleotide protein (RNP) antibody, family history of systemic lupus erythematosus (SLE), she was diagnosed with MCTD for which she followed with rheumatology and was started on azathioprine and hydroxychloroquine (she self-discontinued both medications several months prior to admission out of concern for immunosuppression contributing to her recurrent abscesses). In 2018 she developed sepsis from lymphadenitis. A lymph node biopsy showed chronic inflammation with granulomatous features and serological detection of microbial cell-free DNA of *M. pneumoniae.* Warthin-Starry stain, Grocott-Gomori methanamine silver (GMS), and acid-fast bacilli stains (AFB) were negative.

Her vaccinations were up to date, including a pneumococcal conjugate vaccine (PCV-13) she had received following her splenectomy for ITP. She affirmed a strong family history of lupus and thyroid disease.

She initially met systemic inflammatory response syndrome (SIRS) criteria due to fever of 101.4°F and tachycardia. Physical exam revealed an atraumatic neck with diffuse cervical lymphadenopathy and three abscesses in the submental, supra-jugular, and right lateral posterior neck. She underwent incision and drainage and empiric treatment for *M. pneumoniae* with IV azithromycin. Her white blood cell count was within normal limits, but C-reactive protein was elevated at 5.86 mg/dL (range < 0.5). Gram stain and multiple wound cultures and blood cultures were negative. Broad range PCR of the lymph node aspirate was again positive for *M. pneumonia.*

Given her known history of recurrent and prolonged mycoplasma infections, her mycoplasma IgM and IgG antibodies were measured to rule out primary immunodeficiency. Both were revealed to be negative, raising concern for inability of memory B-cell differentiation. Further immunological workup revealed IgG = 541 mg/dL (reference laboratory range 552–631), IgA = 83 mg/dL (range 65–421), and IgM = 21 mg/dL (range 33–293); her hypogammaglobulinemia pointed toward possible underlying CVID. Her absolute T cell count = 875/mm^3^ (range 603–2990), CD4 helper = 540/mm^3^ (range 441–2156), CD8 = 319 (range 125–1,312), CD19 B cell = 150 (range 107–698/mm^3^), and CD16/56 natural killer cells = 66/mm^3^ (range 95–40). Her pneumococcal vaccine titers were measured, which were low for 11/23 serotypes, solidifying the diagnosis of CVID. She subsequently received intravenous immunoglobulin (IVIG).

Her remaining hospital course was unremarkable. She was discharged on oral azithromycin with a referral to Allergy and Immunology (A&I) outpatient.

Shortly after discharge she returned to the hospital with a similar presentation, again underwent incision and drainage (I&D) by surgery, received IVIG, and was prescribed levofloxacin. Immunoglobulin levels were followed, and IgG initially was within normal limits, but continued IVIG treatment was delayed due to insurance. IgG levels trended down and during this time, and her lymphadenopathy increased in size and number, for which doxycycline was added. IgA levels remained normal and IgM levels remained persistently low. She has since received regular IVIG treatments, with her IgG levels responsive to treatment (IgG = 966 range 552–631, IgA = 79 range 65–421, and IgM = 13 range 33–293). Her pneumococcal vaccine titers have also since normalized.

## Discussion

CVID, as the second-most common primary immunodeficiency, presents with a range of manifestations [1]. Skin sequelae are no exception: skin infections occur nearly four times as often in patients with CVID as compared to the general population, with diverse etiologies, including bacterial, viral, and fungal. Patients may also develop autoimmune diseases, such as vitiligo, alopecia areata, and psoriasis [2–4]. Our case adds to this ever-expanding literature of unique presentations of CVID by being the first of our knowledge to describe *M. pneumoniae* skin abscess in a patient with CVID. We will first discuss the diagnosis and management of CVID, then the broad manifestations with which *M. pneumoniae* can present.

Multiple diagnostic criteria for CVID exist, which we refer to here [5–7]. Generally, these include: age greater than four, evidence of autoimmunity or recurrent bacterial infections, hypogammaglobulinemia (IgG and IgA and/or IgM), poor antibody response to vaccinations, and no other identifiable cause for hypogammaglobulinemia. Moreover, involvement of multiple organ systems in CVID may lead to granulomatous inflammation, interstitial lung disease, autoimmune and hematologic disorders, and increased incidence of lymphocytic malignancy [8]. With the exception of malignancy, our patient had all of the aforementioned criteria. Her history of recurrent infection with mycoplasma led us to suspect immunodeficiency and to test her mycoplasma antibody titers, which were negative. *M. pneumoniae* IgM and IgG should remain positive for several months during and after infection [9, 10]. Additionally, despite multiple abscesses, the patient’s C-reactive protein was only minimally elevated.

With regards to the hypogammaglobulinemia criterion, IgG is traditionally at least two standard deviations below normal range in diagnoses of CVID. Another differential diagnosis for our patient included isolated IgG deficiency, which has a separate *International Classification of Diseases* code (D80.3) from CVID (83.9). An analysis by *Filion *et al*.* comparing two cohorts of patients with CVID and IgG deficiency found that, in addition to markedly low IgG, patients with CVID demonstrated significantly poorer vaccination response to the 23-valent pneumococcal polysaccharide vaccine (our patient was protected against 1/12 serotypes in the Prevnar-13 vaccine, which she had received along with the pneumococcal polysaccharide vaccine three years prior to presentation) and higher rates of autoimmune disease (MCTD in our patient), autoimmune cytopenia (ITP), and granulomatous lesions (pulmonary granulomas) [11]. Although the immunoglobulin titers were higher than what one traditionally expects in a patient with CVID, our patient’s comorbidities correlated to those more commonly found in CVID rather than IgG deficiency. This discrepancy attests to the necessity of thoroughly exploring the possible manifestations of CVID when differentiating between the two diagnoses.

The management of CVID consists of immunoglobulin replacement therapy [12]. Its mechanism in treating CVID has traditionally been thought of as replacement therapy, although recent evidence suggests that replacement also induces proliferation and immunoglobulin synthesis in B cells, rectifying the defunct immunoglobulin synthesis pathway in these patients [13]. In our patient, she reported improved wound healing and decreased malaise following her initial treatment in the hospital. Active infections may be treated with antibiotic therapy, as in our patient, wherein her persistent abscesses were treated with continued fluoroquinolone and tetracycline therapy. Patients should also avoid live vaccines and, due to their increased risk of malignancy, receive all age-appropriate cancer screenings.

With regards to *M. pneumoniae* infections, the most common location in CVID and non-CVID patients is in the respiratory tract [14, 15]. Rarer presentations of *M. pneumoniae* in the general population include central nervous system, cardiac, and cutaneous involvement [16, 17].

Several dermatologic manifestations of *M. pneumoniae* have been described. These include non-specific urticarial and maculopapular rashes, erythema multiforme, Stevens-Johnson syndrome, and *M. pneumoniae*-induced rash and mucositis [18]. The mechanisms of these skin manifestations are thought to be via direct inoculation, secondary immune-mediated damage, vascular occlusion, or direct invasion of the vascular tissue, depending on the presentation [10, 19]. Some manifestations for example, such as the bullous lesions, are thought to be due to hematogenous transfer of the bacteria to the dermis, with damage caused by generation of inflammatory cytokines [10].

Prior to this admission, our patient had previously demonstrated positivity of *M. pneumoniae* abscesses, implying direct invasion of the cutaneous tissue. Her negative culture during our admission points to secondary immune damage or hematogenous transfer from prior infections as the likely mechanism. Our patient had a normal white blood cell count throughout her admission, although leukopenia occurs in 25% of patients with CVID, making hematogenous transfer from one of her prior infections still a possible explanation for the positivity of her *M. pneumoniae* abscesses [20]. A limitation of this case and the patient’s diagnosis is the patient’s prior history of rituximab, which has been documented to cause prolonged hypogammaglobulinemia in up to 30% of cases [21].

## Conclusion

In summary, this clinical vignette describes an unusual case of CVID manifesting with mycoplasma skin abscesses. Diagnosis was aided by her history of autoimmunity and recurrent infection, prompting suspicion of hypogammaglobulinemia and appropriate workup. This case serves as a reminder of the broad symptom manifestations of CVID and diverse diagnostic criteria clinicians should use to diagnose the syndrome. The important teaching point of this case is that, with such a variable presentation, CVID is an important diagnosis to keep on the differential for patients presenting with unexplained disease processes involving the aforementioned signs and symptoms.

## Data Availability

Not applicable.

## Abbreviations

CVID: Common variable immunodeficiency
MCTD: Mixed connective tissue disease
ITP: Immune thrombocytopenia
IVIG: Intravenous immunoglobulin
A&I: Allergy & immunology
IgA: Immunoglobulin A
IgE: Immunoglobulin E
IgG: Immunoglobulin G
GMS: Grocott-Gomori methanamine silver
AFB: Acid-fast bacilli
PCV-13: Pneumococcal conjugate
SIRS: Systemic inflammatory response syndrome
IgM: Immunoglobulin M
